# Price-Cutting Trends in New Drugs after Listing in South Korea: The Effect of the Reimbursement Review Pathway on Price Reduction

**DOI:** 10.3390/healthcare8030233

**Published:** 2020-07-26

**Authors:** Sungju Kim, Jong Hyuk Lee

**Affiliations:** 1 Healthcare Group, Lee & Ko, Seoul 04532, Korea; sungju.kim@leeko.com; 2 Department of Pharmaceutical Engineering, College of Life and Health Sciences, Hoseo University, Asan 31499, Korea

**Keywords:** price cut, post-listing management, reference price, weight average price, pharmaco-economic study, cost-effectiveness

## Abstract

This study aims to analyze the trends of post-listing price changes for new drugs listed from 2007, when the health technology assessment (HTA) was introduced in South Korea, until 2017. We analyzed 135 products that have undergone price cuts. These products were analyzed by their respective review pathways, namely, pharmaco-economic study (PE), weighted average price (WAP), and the without a cost-effectiveness (CE) pathway. Prices were discounted faster in PE than in WAP (*p* = 0.002 in a comparison between PE and WAP). In addition, the median discount rate of the first price cut was 5.0% (range: 0.1–20.0) for PE, 3.0% (range: <0.1–30.0) for WAP, and 5.0% (range: 0.6–10.9) without a CE pathway. The median cumulative discount rate of PE and WAP showed that the PE pathway products’ discount rates were higher: 10.4% for PE and 6.0% for WAP (*p* = 0.025 for comparison between PE and WAP). It is necessary to discuss the practical effects of the price-cutting system from a myriad of perspectives, including insurance finance, the value of new drugs, and the accessibility of new drugs to patients.

## 1. Introduction

With improvements in medical technology and biotechnology, innovative new drugs, such as cell therapy products and immunotherapeutic products, including monoclonal antibody drugs, are being developed continuously. Developing new drugs takes considerable time and has a high risk of failure, as a drug faces many barriers before becoming a successful blockbuster [1,2,3,4]. In particular, reimbursement and pricing processes, which are crucial stages for market access and patient access, are hurdles in the final stage of commercialization [5,6,7,8,9]. In other words, pharmaceutical companies have to go through many hurdles to recoup research and development (R&D) expenses spent on developing new drugs and invest in R&D of other new drugs. In this situation, patients can also benefit from taking advantage of innovative new drugs to treat their disease, which makes for a virtuous cycle [3]. However, the payer, who administers public insurance, has to take the effective management of budgets into account, owing to the financial burden caused by an increase in pharmaceutical expenditure [9,10,11,12,13,14]. In other words, the health technology assessment (HTA) body, which ought to efficiently distribute limited resources regarding the reimbursement and pricing of new drugs, needs to consider not only patients’ access, but also the budget impact of new drugs. In this context, it is natural for stakeholders’ stance related to new drugs to be contradictory. Orphan drugs, anti-cancer drugs, and cell/genetic therapy products, in particular, are required by very few patients; therefore, these have a small potential market. In this situation, when the price is high, such problems may cause more serious issues. High-price drugs are often reimbursed because of social necessity even if the clinical outcome is uncertain; however, an unexpected increase in patient numbers may have a considerable impact on budgets [10,11,12,13,14,15,16]. Pharmaceutical companies need to raise prices to generate expected profits, while governments become more cautious about whether to reimburse or not, due to the financial burden of allocating a large amount of money to a small number of patients. In this situation, delayed or failed reimbursements cause problems related to patient access. Therefore, in terms of the determination of related policies, securing procedural justification that reflects different stakeholders’ stances (pharmaceutical company, government, and patients) is highly important. In major countries implementing HTA, decision-making regarding the pricing of new drugs is a crucial issue; they are also carrying out policies to ensure patient access such as risk sharing agreements (RSA) in reimbursement in decision-making and conditional reimbursements [17,18,19]. In addition, major countries are also implementing policies, such as price-volume agreements (PVA), and re-evaluating pricing methods to manage financing after the listing of new drugs [20,21,22]. For the cost containment of new drugs, numerous countries use internal/external reference prices (ERP) in listing as crucial factors when determining the price of newly developed drugs [23,24,25,26,27]. In other words, a new drug’s price in one country affects other countries’ drug price structures, which makes stakeholders sensitive to decision-making regarding the pricing of new drugs. Therefore, both the listing price of new drugs and the price change after listing have become highly important factors.

South Korea has implemented HTA, and rigorously regulates the reimbursement of listed prices through cost-effectiveness evaluations and negotiations. South Korea also implemented post-listing pricing control system to reduce prices by various mechanisms, even after a drug is listed. Therefore, in South Korea, not only the list price of new drugs, but also the post-listing price change may affect other drugs’ pricing, through internal and external reference prices. In other words, if the number of price cuts increases by post-listing management in South Korea, the price affects pricing in other countries, as well as the price of new drugs with similar efficacy in South Korea. In addition, price changes directly affect the patient’s co-payment and insurance finances. The post-listing price change of new drugs is recognized as a significant issue for all stakeholders, and policy decision-making regarding this issue can be influenced by a number of variables. Securing transparency and consistency so that stakeholders can predict what will happen next is also important. To achieve the policy objective, policymakers should ensure receptivity through social consensus. Since some new drugs have excessively low prices, which affects ERP, at the price determination stage, some pharmaceutical companies give up on selling such drugs in South Korea [28,29].

The price of new drugs is an important factor, because of the interests of various stakeholders in South Korea, but very few studies have focused on post-listing price cuts. In South Korea, prices fluctuate due to various causes after listing, but there is no actual research on this phenomenon. Considering various perspectives, this study analyzed trends in post-listing price changes for all new drugs listed from 2007, when HTA was introduced into Korea, to 2017, over a period of 11 years in total.

### 1.1. New Drug Pricing and Reimbursement Review System in South Korea

In the South Korean HTA system, the reimbursement of new drugs and the review of prices follow three pathways, which are differentiated by the methods used to verify the cost-effectiveness. The first is applied when the drug has superior clinical usefulness compared to its alternatives. When cost-effectiveness is verified via a pharmaco-economic (PE) study, the drug will be reimbursed at a higher price compared to the internal reference price of the alternative (PE pathway). The second is applied when the drug has superior or non-inferior clinical usefulness, in which case the drug is reimbursed at a price below the weighted average price (WAP) of the alternative (WAP pathway). The third method is applied to life-threatening diseases, such as rare diseases or cancer, when there is no alternative drug or a low number of potential patients, so the verification of cost-effectiveness is impossible. The determination of reimbursement is based on the price in seven countries (the so-called A7 countries: USA, Japan, Germany, France, Switzerland, UK, and Italy) as the external reference price without cost-effectiveness verification (without CE pathway). In this case, RSA may be applied to spread financial risk related to uncertain clinical usefulness and budget impact [30,31].

### 1.2. Post-Listing Price-Cutting System in South Korea

The post-listing price-cutting system can be classified into four types: PVA, transaction price policy, price cutting with expanded indication, and price cutting of patent-expired originals and generics. Since discounts based on the four mechanisms can be duplicated, a drug may go through several price cuts.

#### 1.2.1. PVA

When a new drug is listed, the estimated annual amount will be negotiated between pharmaceutical companies and the Korean National Health Insurance Service (NHIS). When the annual amount is exceeded by 30%, the drug price will be reduced by up to 10% by price-cutting negotiations. Consequently, the amount of expenditure will be monitored annually. The price will be reduced again by up to 10% of the price when expenditure increases by 60% or more from the previous year, or when the expenditure increases by 10% and more than KRW 5 billion [32,33].

#### 1.2.2. Transaction Price Policy

Based on a biennial investigation into the actual transaction price of a new drug, the price can be discounted by up to 10% when the actual transaction price is lower than the reimbursement price [32,33].

#### 1.2.3. Price Cutting with Expanded Indication

For drugs with an expanded indication, where the expected increase in claim amounts are between KRW 1.5 billion and KRW 10 billion, a price cut is implemented by applying a discount rate derived from the expected additional claims and the expected increase in the rate of claims [32,33]. The discount rate of the cap can be applied from 1% to 5%; the rate is applied based on the price when the scope of the reimbursement is extended. When an increase of more than 10 billion KRW is expected, the discount rate (1% to 5%) is not applied, and the price cut is established by price-cutting negotiation [33].

#### 1.2.4. Price Cutting of Patent-Expired Originals and Generics

After the expiration of a patent, the drug’s original price is reduced by 70% and the price of generic drugs by 59.5% for one year to promote stable supply and the faster listing of the generic drug when a generic drug is listed. However, after one year has passed since the generic drug is listed, the price will be discounted to 53.55% of the price of the first branded drug, regardless of the original or generic drug [32,33].

## 2. Materials and Methods 

This study analyzed price changes between new drugs’ listed date—new drugs listed from 1 January 2007 to 31 December 2017 (hereinafter referred to as the listing period)—and 31 July 2019 (hereinafter referred to as the price monitoring period), in other words, over a period of 11 years. In total, the price cuts and price-cutting history by year of 135 drugs that have undergone price cuts were analyzed and categorized by review pathway at the time of the listing (PE, without CE, and WAP pathway). Products that do not have a price cuts were censoring data, but there were many products (e.g., orphan drugs, products with an annual total claim amount of less than 1.5 billion KRW) that were excluded from the price cut in post-listing price cut system. Furthermore, as we could not obtain the information about excluded products, these were excluded from the analysis. In addition, the price-cutting rate by year, and the time of the first price cut and its rate were analyzed by review pathway.

Descriptive statistics (median, minimum, and maximum) were used to analyze the discount rate. Regarding the drug price discount rate by review pathway and the timing of the first price cut and its rate, the Mann-Whitney U test was used, because these data should be right-skewed; the subject was the PE pathway, and the WAP pathway (without CE pathway was excluded), because it was judged to be important to test the statistical difference between the main listing pathways. Analyses were conducted using SPSS version 19.0 (SPSS Inc. Chicago, IL, USA). All *p*-values were two-tailed, and *p* < 0.05 was considered statistically significant. The list of newly listed drugs and price change data were obtained from publicly disclosed data from the Health Insurance Review and Assessment Service’s website, the Korean Ministry of Food and Drug Safety’s website, and the Ministry of Health and Welfare’s website. 

## 3. Results

### 3.1. Categorization by Review Pathway of New Drugs

The results of the categorization of 198 new drugs listed during the listing period are as follows: 54 (27.3%) had gone through the PE pathway, 21 (10.5%) without CE, and 123 (62.1%) through the WAP pathway. The proportion of WAP was the highest. Of those products, 135 products were discounted (135/198, 68.2%). The 135 products were categorized by their review pathway, of 135 which 43 were PE (43/54, 79.6%), 9 were without CE (9/21, 42.9%) and 83 were WAP (83/123, 67.5%). The share of the PE pathway was the highest. Meanwhile, the 63 drugs that were not discounted were categorized by their review pathway, of which 11 were PE (11/54, 20.4%), 12 were without CE (12/21, 57.1%) and 40 were WAP (40/123, 32.5%) (Table 1).

### 3.2. The Pricing Cutting Trend Based on the Four Mechanisms 

The result of the analysis regarding the cut history for each price cutting mechanism showed that 91 products were cut by PVA, a median cutting rate was 4.6% (range: 0.03–9.4), and 50 products, by price cutting with expanded indication, a median rate was 5.0% (range: 0.5–57.7), and 78 products, by transaction price policy, a median rate was 0.3% (range: 0.1–8.1). A total of 25 products were cut by the price cutting system of patent-expired originals.

### 3.3. Time Elapsed Until the First Price Cut Since Listing and Its Rate by Review Pathway 

The result of the analysis regarding the time elapsed before the 135 products received their first price cut after listing showed that PE took 24.0 months (range: 4.0–60.0), without CE took 22.0 months (range: 12.0–83.0), and WAP took 34.0 months (range: 2.0–88.0). The price was discounted faster in the PE group than in the WAP group; the difference was statistically significant (*p* = 0.002 for comparison between PE and WAP). In addition, the median discount rate of the first price cut was 5.0% for PE (range: 0.1–20.0), 3.0% for WAP (range: <0.1–30.0), and 5.0% without CE (range: 0.6–10.9). While the discount rate of the PE group was the highest, there was no statistically significant difference (Table 2) (*p* = 0.177 for comparison between PE and WAP). 

### 3.4. Number of Price Cuts and Median Cumulative Discount Rates for New Drugs

An analysis of the number of discounts and median cumulative discount rate for the 135 products showed that the number of products discounted more than twice was 91 (91/135, 67.4%); one product was discounted six times. The median cumulative discount rate of the products discounted only once was 2.2% (range: <0.1–30.0); the median cumulative discount rate of the 135 products was 6.5% (range: <0.1–63.6) (Table 3). The median cumulative price cut rate of products which were discounted four times jumped, due to price cutting of patent-expired products.

### 3.5. Price-Cutting Rate of New Drugs after Listing by Year

The median cumulative discount rate of the 135 products that were discounted within the price monitoring period was 4.5% (range: <0.1–63.6) in the third year, and 5.6% (range: <0.1–58.1) in the fifth year. In other words, 90 (90/123, 73.2%) and 79 (79/87, 90.8%) products showed a median price discount of 4.5% after 3 years of listing and 5.6% after 5 years of listing, respectively. In the 10th year, 18 products were discounted by a median of 10.1% (range: 0.3–50.3) (Table 4).

### 3.6. Comparative Analysis of Discount Rates of Drug Prices According to Listed Pathways by Year

**Figure 1:**
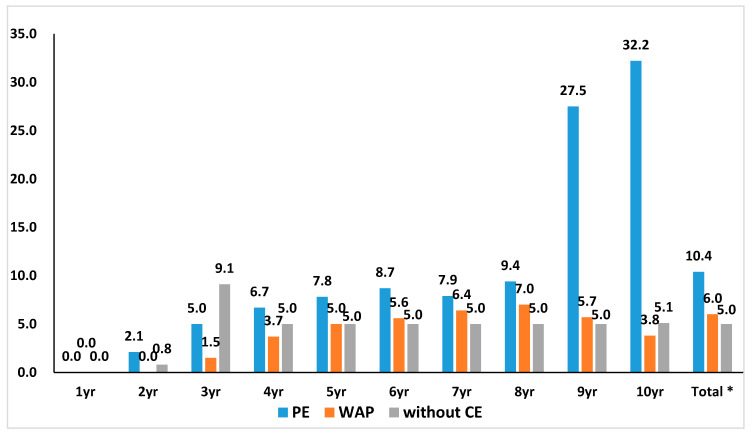
Cumulative price cut rate by review pathway at the time of new drug’s listing by year. The median cumulative discount rate of 135 products by review pathway and year showed that the discount rate in the pharmaco-economic study (PE) was highest in general. * A comparison of PE and weighted average price (WAP) showed that PE products’ discount rate was higher (10.4% for PE, 6.0% for WAP). The difference showed a statistical significance. (*p* = 0.025 for comparison between PE and WAP).

The result of the analysis of the median cumulative discount rate of the 135 products by their review pathway and year showed that the discount rate for PE was the highest. A comparison of the median cumulative discount rate of PE and WAP showed that the PE pathway products’ discount rate was higher (10.4% for PE study, 6.0% for WAP). The difference showed a statistical significance (Figure 1) (*p* = 0.025 for comparison between PE and WAP). 

## 4. Discussion

This study analyzed the drug price discount trends of new drugs listed in South Korea over the past 11 years (2007 to 2017). Of the 198 listed drugs, the prices of 123 (62.1%) drugs were determined by referencing the internal reference price (WAP pathway). In other words, post-listing discounts have a significant effect on subsequent development and new drugs. In addition, of the new drugs listed, the prices of 135 (68.2%) drugs were discounted, and the prices of 91 (67.4%) drugs were discounted more than twice. On median, prices were discounted by 5.6% (<0.1–58.1) after five years had passed after listing and by 10.1% (<0.1–50.3) after 10 years had passed after listing. The prices of new drugs are discounted through various mechanisms. However, uncertainty related to price change is high, because the timing, number, and rate of discount are hard to predict. In this vein, companies that develop new drugs may make a case that price predictability for new drugs is low, while the payer may experience a problem in predicting the pharmaceutical expenditure. In particular, domestic and foreign companies that develop new drugs are highly sensitive to drug prices, since the South Korean price actually affects pricing in foreign countries through ERP.

This study also confirmed that the new drugs listed via the PE pathway, drugs listed at the higher price of alternatives’ WAP, showed a faster and larger price discount rate. The payer may consider this effective fiscal management, since new drugs that had maintained a higher price to its alternatives are discounted. In fact, price cut occurred most frequently by PVA and price cutting with expanded indication, and the average cutting rate is also very high. In these two price cutting systems, the price cutting rate is determined by negotiations between the pharmaceutical company and NHIS. In the end, we can assume that NHIS has led to determine high cut rates through the negotiation for products that are listed by the PE pathway, to alleviate financial burdens. However, some may argue that discounting the price of new drugs more frequently and at a faster rate, without considering cost-effectiveness after listing, even if the drugs proved cost-effective at the time of listing, can hinder consistency in the mechanism of listing new drugs and post-listing management. In addition, since not only price, but also usage, greatly affects overall pharmaceutical expenditure, a method that considers managing usage is important for effective fiscal management.

For the payer, to ensure the adequateness of the listing price of new drugs, and especially to cope with the high pricing strategies of pharmaceutical companies, who maintain a monopoly hold on new drugs they develop, a policy that prioritizes patient accessibility at the listing stage, and then manages finance through price discounting in the post-listing stage, is required. However, the actual effect of such discounts on new drugs has not been comprehensively evaluated.

In South Korea, the stakeholder’s position related to price cutting after the listing of new drugs is in conflict. Pharmaceutical companies claim that, despite the low listing price of new drugs, the price was excessively cut after listing. Direct control of drug prices is a powerful means to secure the financial stability of health insurance programs, and can be an effective policy when it is used properly. However, previous studies argued that such policies may have no actual effect due to the increase in the volume of drug usage [34,35,36,37]. In this vein, additional research regarding the effectiveness of post-listing management policy focusing on price is needed. However, the government argues that the price cut should be increased, because the listing price of new drugs is high, and the price cut after listing is low, which has a great impact on the national health insurance budget.

This study has some limitations, because we intended to show the status of the price change using descriptive statistics rather than statistical tests. There was a time limitation to confirm cumulative discount rate completely. We can see a trend that more products were listed under PE pathway up to 2017. Consequently, we only had incomplete data to confirm the discount rate since those products that were listed more recently, such as, in 2015, did not have the 10th year’s data.

While this study analyzed price changes after the listing of new drugs, an analysis of the impact of the variables (the competition, product substitution, therapeutic groups, innovativeness of the product, etc.) regarding its actual effect, could not be performed, because the scope of research was too extensive and data were not available. 

Nevertheless, stakeholders such as pharmaceutical companies trying to release new drugs in Korea, and policy decision-makers managing the finance of the insurance programs may take advantage of this research as a reference for financial decision-making, including post-listing price prediction. In addition, nations operating HTAs similar to South Korea may utilize this research by referring to it for policy decision-making for reimbursement, pricing, post-listing drug price management, or related additional research.

## 5. Conclusions

In the South Korean health insurance system, four mechanisms are used to calculate discount rates after the listing of new drugs. Since the same product may be discounted multiple times, uncertainty relating to post-listing price changes is high. Moreover, new drugs listed through the PE pathway have a higher discount rate and are discounted faster. In other words, the rapid loss of new drugs’ value, which was appreciated at the listing stage, may benefit the payer and patients since pharmaceutical expenditure can be lowered. However, for pharmaceutical companies, such a mechanism will be considered as a risk: consequently, companies may shy away from developing and listing new drugs, and this may eventually lower patients’ accessibility to new drugs. While there is no basis for clearly selecting an adequate amount of time after which an evaluated price at the initial listing can be changed, it is possible to evaluate whether the post-listing management of the drug price is being effectively operated according its original purpose. In other words, it is necessary to discuss the practical effects of the drug cost reduction policy from a myriad of perspectives, such as, not only insurance finance, but also the value of new drugs, and its accessibility to patients.

## Figures and Tables

**Table 1:** Categorization of new listed drugs by review pathway (2007–2017).

Year	No. of New Listed Drugs	Review Pathway (No. of Product)		
		PE	Without CE	WAP
2007	1	1	0	0
2008	8	4	1	3
2009	15	3	2	10
2010	15	4	2	9
2011	12	2	0	10
2012	20	2	1	17
2013	17	5	1	11
2014	18	7	0	11
2015	34	9	2	23
2016	22	6	5	11
2017	36	11	7	18
Total (%)	198 (100)	54 (27.3)	21 (10.5)	123 (62.1)
Discounted products (%)	135 (68.2)	43 (79.6)	9 (42.9)	83 (67.5)
Non-discounted products (%)	63 (31.8)	11 (20.4)	12 (57.1)	40 (32.5)

PE: Pharmaco-Economic study; CE: Cost-Effectiveness; WAP: Weighted Average Price.

**Table 2:** Time elapsed until the first price cut after listing and its rate by review pathway.

PE			Without CE			WAP		
No. of Product	Median Time to First Cut	Median Price Cut Rate	No. of Product	Median Time to First Cut	Median Price Cut Rate	No. of Product	Median Time to First Cut	Median Price Cut Rate
	Month	%		Month	%		Month	%
43 (31.9%)	24.0 * (4.0–60.0)	5.0 ** (0.1–20.0)	9 (6.6%)	22.0 (12.0–83.0)	5.0 (0.6–10.9)	83 (61.5)	34.0 * (2.0–88.0)	3.0 ** (<0.1–30.0)

PE: Pharmaco-Economic study; CE: Cost-Effectiveness; WAP: Weighted Average Price; SD: Standard Deviation. * Statistically significant (*p* = 0.002 for comparison between PE and WAP). ** No statistically significant difference (*p* = 0.177 for comparison between PE and WAP).

**Table 3:** Number of price cuts and median cumulative price cut rates of new drugs.

No. of Price Cuts	No. of Products	Ratio (%)	Median Cumulative Discount Rate (%) (Min–Max)
1	44	32.6	2.2 (<0.1–30.0)
2	43	31.9	5.9 (0.1–63.6)
3	28	20.7	9.6 (0.3–53.4)
4	13	9.6	49.0 (13.5–58.1)
5	6	4.4	36.5 (15.2–54.4)
6	1	0.7	39.3 (NA)
Total	135	100	6.5 (<0.1–63.6)

**Table 4:** Cumulative price-cutting rate of new drugs after listing by year.

Years *	Products **	No. of Product Price Cuts	Ratio (%)	Median Cumulative Price-Cutting Rate (%) (Min–Max)
1 yr.	135	10	7.4	6.3 (0.5–27.9)
2 yr.	132	53	40.2	5.5 (<0.1–27.9)
3 yr.	123	90	73.2	4.5 (<0.1–63.6)
4 yr.	103	90	87.4	5.5 (<0.1–49.5)
5 yr.	87	79	90.8	5.6 (<0.1–58.1)
6 yr.	75	72	96	6.2 (<0.1–54.2)
7 yr.	55	50	90.9	6.4 (<0.1–49.7)
8 yr.	38	38	100	7.0 (0.1–50.3)
9 yr.	29	29	100	6.6 (0.1–50.3)
10 yr.	18	18	100	10.1 (0.3–50.3)

* Based on the month of listing, one year is calculated as a period of 12 months after the listing. ** Even if a product was discounted in the first year and did not get discounted in the second and third year, the product will still be included in the number of products in the second and third year.
